# Identification of a novel cathelicidin antimicrobial peptide from ducks and determination of its functional activity and antibacterial mechanism

**DOI:** 10.1038/srep17260

**Published:** 2015-11-26

**Authors:** Wei Gao, Liwei Xing, Pei Qu, Tingting Tan, Na Yang, Dan Li, Huixian Chen, Xingjun Feng

**Affiliations:** 1 College of Animal Science and Technology, Northeast Agricultural University, Harbin 150030, P.R. China

## Abstract

The family of antimicrobial peptide, cathelicidins, which plays important roles against infections in animals, has been identified from many species. Here, we identified a novel avian cathelicidin ortholog from ducks and named dCATH. The cDNA sequence of dCATH encodes a predicted 146-amino-acid polypeptide composed of a 17-residue signal peptide, a 109-residue conserved cathelin domain and a 20-residue mature peptide. Phylogenetic analysis demonstrated that dCATH is highly divergent from other avian peptides. The α-helical structure of the peptide exerted strong antimicrobial activity against a broad range of bacteria *in vitro*, with most minimum inhibitory concentrations in the range of 2 to 4 μM. Moreover, dCATH also showed cytotoxicity, lysing 50% of mammalian erythrocytes in the presence or absence of 10% fetal calf serum at concentrations of 32 μM or 20 μM and killing 50% HaCaT cells at a concentration of 10 μM. The effects on bacterial outer and inner membranes, as examined by scanning electron microscope and transmission electron microscopy, indicate that dCATH kills microbial cells by increasing permeability, causing a loss of membrane integrity.

In previous decades, the development of multiple antibiotic resistant bacteria has become a growing global public health problem that has hastened an aggressive search for novel antimicrobial agents with little structural similarity to those conventional antibiotics^1^. Antimicrobial peptides (AMPs) have been acknowledged as potential novel pharmaceutical agents due to their broad-spectrum antimicrobial activity and reduced bacterial resistance^2^. Cathelicidins are a major family of AMPs newly identified in vertebrate animals^3^. Generally, this series of peptides typically contain a conserved N-terminal sequence (“cathelin” domain) and a C-terminal antimicrobial domain of varied sequence and length that is a highly diversified and biologically active mature sequence^4^.

Cathelicidins have been found in almost all types of vertebrates, where they play vital roles in the immune system^5^. Upon activation, most cathelicidins are proteolytically cleaved to release the cathelin domain and C-terminal mature peptides with antimicrobial activities^6^. In addition to their primary antimicrobial activities against bacteria, fungi, and enveloped viruses, mature cathelicidins play vital roles in various phases of the host immune system, such as induction of angiogenesis; promotion of wound healing; chemotaxis of neutrophils, monocytes, mast cells and T cells; and inhibition of apoptosis^6^.

After the first cathelicidin, namely Bac5, was discovered from bovine neutrophils^7^, then a large number of cathelicidins have been identified from different vertebrate species, including human, monkey, rat, mouse, rabbit, cattle, guinea pig, sheep, goat, horse^8^. And some distinct cathelicidin genes have been reported in birds, such as Fowlicidin1-3^9^ chCATH-B1^10^ and CMAP27 (*Gallus gallus*)^11^, Pc-CATH1-3 (*Phasianus colchicus*)^12^, Cc-CATH1-3 (*Coturnix coturnix*)^13^, CL-CATH2-3 (*Columba livia*)^14^. With the recent completion of genome sequencing of the mallard duck^3^,^15^, two duck cathelicidin gene sequences (Ap-CATH1, Ap-CATH2) only contained partial segments and encoded amino-acid sequences, have been predicted and deposited in GenBank. However, to the best of our knowledge, there are no reports on cathelicidins with the complete amino-acid sequence found in ducks to date.

In the current study, gene cloning and characterization of avian cathelicidin orthologs, namely dCATH in ducks, are reported. Discovery of the cathelicidin peptide will help reveal the origin and evolution of waterfowl cathelicidins. The peptide dCATH was chemically synthesized, and its antimicrobial activity and mechanism were examined. The secondary structure in different buffer environments and membrane-mimicking activity of the peptide were measured. The antimicrobial properties of the peptide were evaluated by determining the minimum inhibitory concentrations (MIC) against a broad selection of threatening microbes, including Gram-negative and Gram-positive bacteria. Then the hemolytic activity and cytotoxicity were measured. Finally, whole bacteria were further employed to investigate potential membrane destruction mechanisms. Scanning electron microscopy (SEM) and transmission electron microscopy (TEM) were used to directly observe changes in cell morphology as a result of peptide treatment.

## Results

### Identification of duck cathelicidin

**Figure 1:**
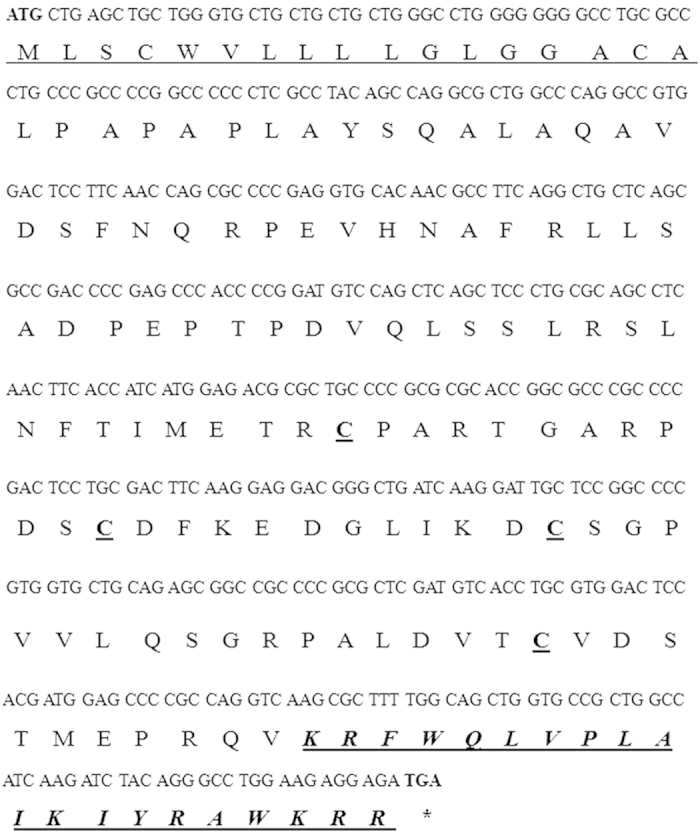
Nucleotide and amino acid sequences for the peptide. Start and stop codons are bold. Signal peptide sequence predicted by SignalP Version 3.0 software (http://www.cbs.dtu.dk/services/SignalP/) is underlined, the four cysteines are bold and underlined. The predicted mature peptide is displayed in bold and italics.

As shown in Fig. 1, one positive clone containing the dCATH cathelicidin cDNA was identified and isolated. The 441-bp cDNA and the deduced 146-amino-acid sequence from the cDNA sequence are shown in Fig. 1, which has been deposited in GenBank (GenBank accession number: KT230679). The N-terminal region contained a classical 17-amino-acid N-terminal signal peptide sequence, as predicted by SignalP software (Version 3.0, http://www.cbs.dtu.dk/services/SignalP/). The remaining propeptide contained a characteristic cathelin-like domain with two conserved cysteine bridges and a C-terminal antimicrobial domain. Given that valine is the optimal protease cleavage site for the maturation of most cathelicidins^16^,^17^, valine at position 126 (Val126–Lys127) is assumed to be the elastase cleavage site, which releases the mature dCATH: KRFWQLVPLAIKIYRAWKRR.

### Phylogenetic tree analysis

**Figure 2:**
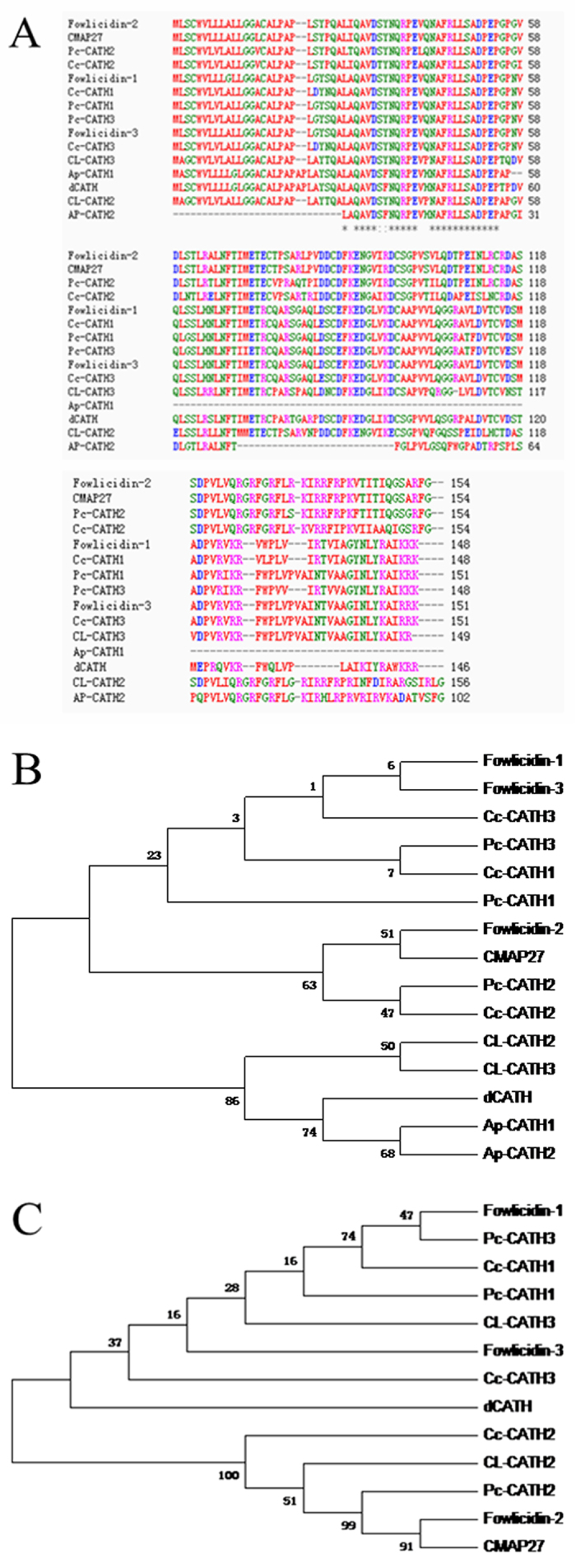
(**A**) Multiple sequence alignment analysis of the duck dCATH with representative avian cathelicidins. dCATH is aligned with classic cathelicidins (CATH1-3 (*Phasianus colchicus*), CATH1-3 (*Coturnix coturnix*), fowlicidin1-3 (*Gallus*), CMAP (*Gallus*), CLCATH2-3 (*Columba livia*), Cathelicidin1-2 (*Anas platyrhynchos*)). (**B**,**C**) Phylogenetic analysis of dCATH and other avian cathelicidins on the basis of the complete peptide (**B**) and mature domain (**C**). The phylogenetic dendrogram was constructed by the Neighbor-joining method based on the proportion difference of aligned amino acid sites of the sequence. Only branches supported by a bootstrap value (expressed as percentage of 1000 bootstrap samples supporting the branch) are shown at the branching points.

The predicted polypeptide was contrasted with classic cathelicidins from different avian species, displayed characteristic features of cathelicidins and showed high similarity (Fig. 2A). The avian multi-sequence alignments were performed based on the proregion and mature domain. Surprisingly, the cathelicidins share a high degree of similarity, particularly in the proregion sequence region, implying that cathelicidins may be more closely related.

Phylogenetic analysis of dCATH and the other avian cathelicidins was also performed on the proregion and mature domain. As shown in Fig. 2B, all avian cathelicidins formed two distinct clusters. *Columba livia* and duck family located in a separated clade from others, so we can predict that they are closely related through the process of evolution. The evolutionary distance indicated that dCATH is more distant but may have a common ancestor compared with others. Between Shaoxing anatis and *Anas platyrhynchos* demonstrated a very little difference, thus, they were considered to be evolutionary “closeness”. As shown in Fig. 2C, there are significant differences in mature sequence between duck and other four species. These differences most likely arise from the fact that duck and the other species live in different environments and gradually formed different AMPs when exposed to different pathogenic microorganisms.

### Structure variability of the peptide in different environments

**Figure 3:**
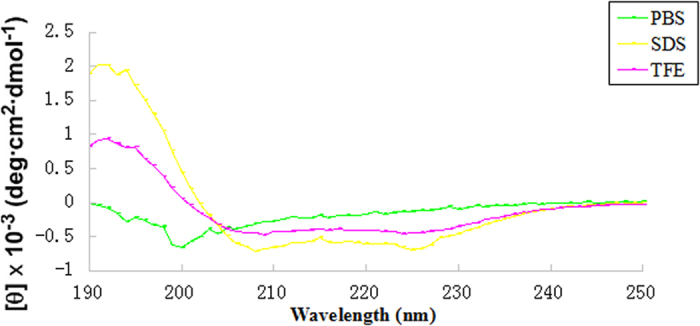
The peptide was dissolved in 10 mM PBS (pH 7.4), 50% TFE, or 30 mM SDS. The mean residue ellipticity was plotted against wavelength. The values from three scans were averaged per sample, and the peptide concentration was fixed at 150 μM.

Circular dichroism (CD) spectroscopy was performed on the peptide in phosphate buffer, 30 mM sodium dodecyl sulfate (SDS), and 50% tetrafluoroethylene (TFE) (Fig. 3). A negative peak is displayed near 200 nm in sodium phosphate buffer, which is a typical feature of a random coil structure of proteins and peptides. Furthermore, the peptide in phosphate buffer showed no secondary helical structure. In TFE and SDS solution, dCATH showed a positive peak band near 192 nm, while in SDS, two negative characteristics of the acromion band were seen at 208 nm and 226 nm, which is typical of an α-helical structure. However, the negative peak was not very obvious in TFE.

### Antimicrobial activities of the peptide

MICs of the synthetic dCATH peptide against Gram-negative and Gram-positive bacteria are presented in Table 1. dCATH exhibited potent antimicrobial activity against all bacterial strains tested, with MICs ranging from 2 to 8 μM. The calculated geometric mean (GM) obtained by MICs for all tested strains in the experiment reflect the therapeutic effect of the peptide against typical bacterial strains in the clinic. The GM (geometric mean) value of dCATH was 4 μM.

### Hemolytic and cytotoxic activity

**Figure 4:**
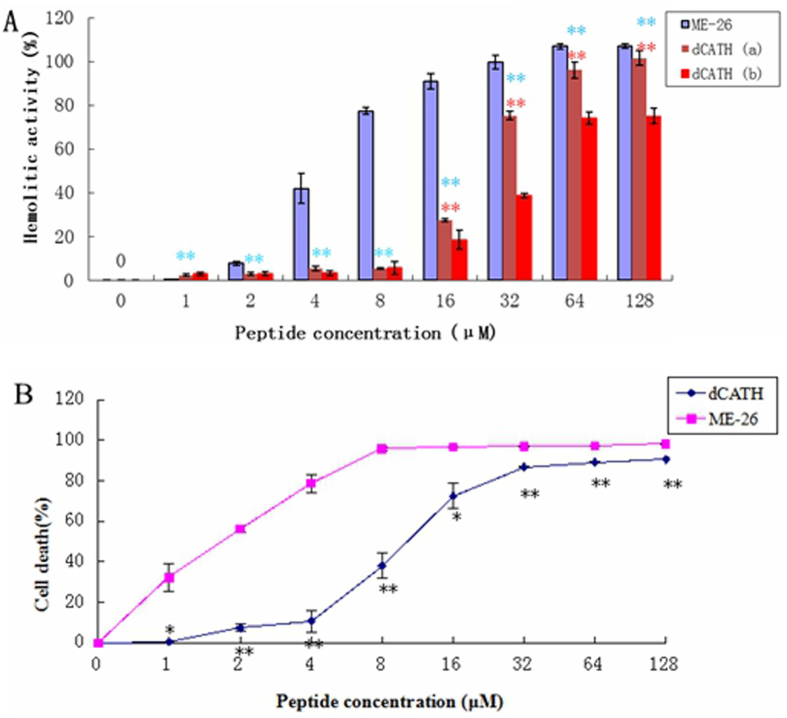
(**A**) Hemolytic activity of the peptides. Hemolytic activity was evaluated by incubating individual peptide in serial 2-fold dilutions with freshly isolated human erythrocytes in the absence (dCATH (a)) or presence (dCATH (b)) of 10% FBS at 37 °C for 2 h, followed by measuring the released hemoglobin at 405 nm. No FBS was added in the hemolytic activity of melittin (*P < 0.05; **P < 0.01; by unpaired t test. The blue * indicates the difference between melittin and dCATH (a), the red ones means the difference between dCATH (a) and dCATH (b)). (**B**) Cytotoxic activity of the peptides. HaCat cells were used to evaluate the toxicity of the peptides to mammalian cells, and measured the released MTT at 492 nm. All the tests were performed three times (*P < 0.05; **P < 0.01; by unpaired t test).

The hemolytic activity of the peptide against human erythrocytes was determined as a measure of toxicity to mammalian cells. Figure 4A showed the hemolytic activity toward human erythrocytes, with 50% killing of mammalian erythrocytes occurring at 20 μM and 32 μM for dCATH in the absence (a) or presence (b) of 10% fetal calf serum (FBS), respectively. In contrast, melittin used as a control peptide caused 50% killing of mammalian erythrocytes occurring at 5 μM. The hemolytic activity of dCATH was reduced in the presence of 10% FBS.

To further examine the cytotoxicity of the peptide towards mammalian epithelial cells, the viability of HaCat cells treated with the peptide was measured. As shown in Fig. 4B, dCATH and melittin addition resulted in a low cell survival rate at high concentrations and 50% viability of HaCaT cells at 10 μM and 1.5 μM respectively.

### OM permeabilization

**Figure 5:**
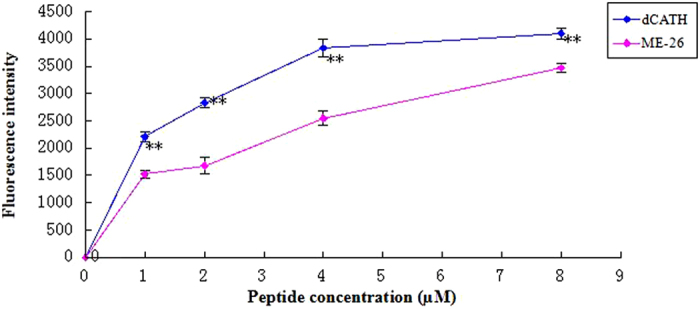
The outer membrane permeability of the peptide dCATH. The outer membrane permeability of *E. coli* UB1005 in the presence of different peptide concentrations (dCATH, ME-26) was determined using the fluorescent dye (NPN) assay. The NPN uptake was monitored at an excitation wavelength of 350 nm and an emission wavelength of 420 nm (*P < 0.05; **P < 0.01; by unpaired t test).

An uptake assay was used to investigate the ability of the peptide dCATH to disturb bacterial outer membrane permeabilization, and *E. coli* UB1005 was used as the model. As shown in Fig. 5, dCATH and melittin were demonstrated to permeabilize the outer membrane of *E. coli* in a dose-dependent manner, as observed by an increase in 1-N-phenylnaphthylamine (NPN) fluorescence. The peptide was able to permeabilize the outer membrane even at concentrations below the MIC. Compared with melittin, dCATH possesses a stronger ability (P < 0.01) to permeabilize the outer membrane at each concentration.

### Cytoplasmic membrane electrical potential

**Figure 6:**
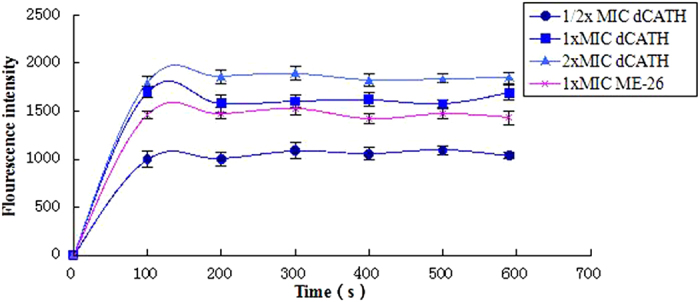
Cytoplasmic membrane electrical potential. The cytoplasmic membrane potential variation of *E. coli* UB1005 treated by peptides (dCATH, ME-26), as assessed by release of the membrane potential-sensitive dye diSC_3_-5.

Upon permeabilization and disruption of the cytoplasmic membrane, the membrane potential is dissipated and 3,3′-Dipropylthiadicarbocyanine iodide (diSC_3_-5) is released into the buffer, which results in an increase in fluorescence that can be detected by fluorescence spectrometry. As shown in Fig. 6, the membrane depolarization was measured after peptide addition. dCATH at its 1× or 2× MIC concentration was more effective and rapid at permeabilizing the membrane than melittin at 1× MIC concentration (P < 0.01). dCATH depolarized the bacterial cytoplasmic membrane in a dose-dependent manner.

### Scanning electron microscopy (SEM)

**Figure 7:**
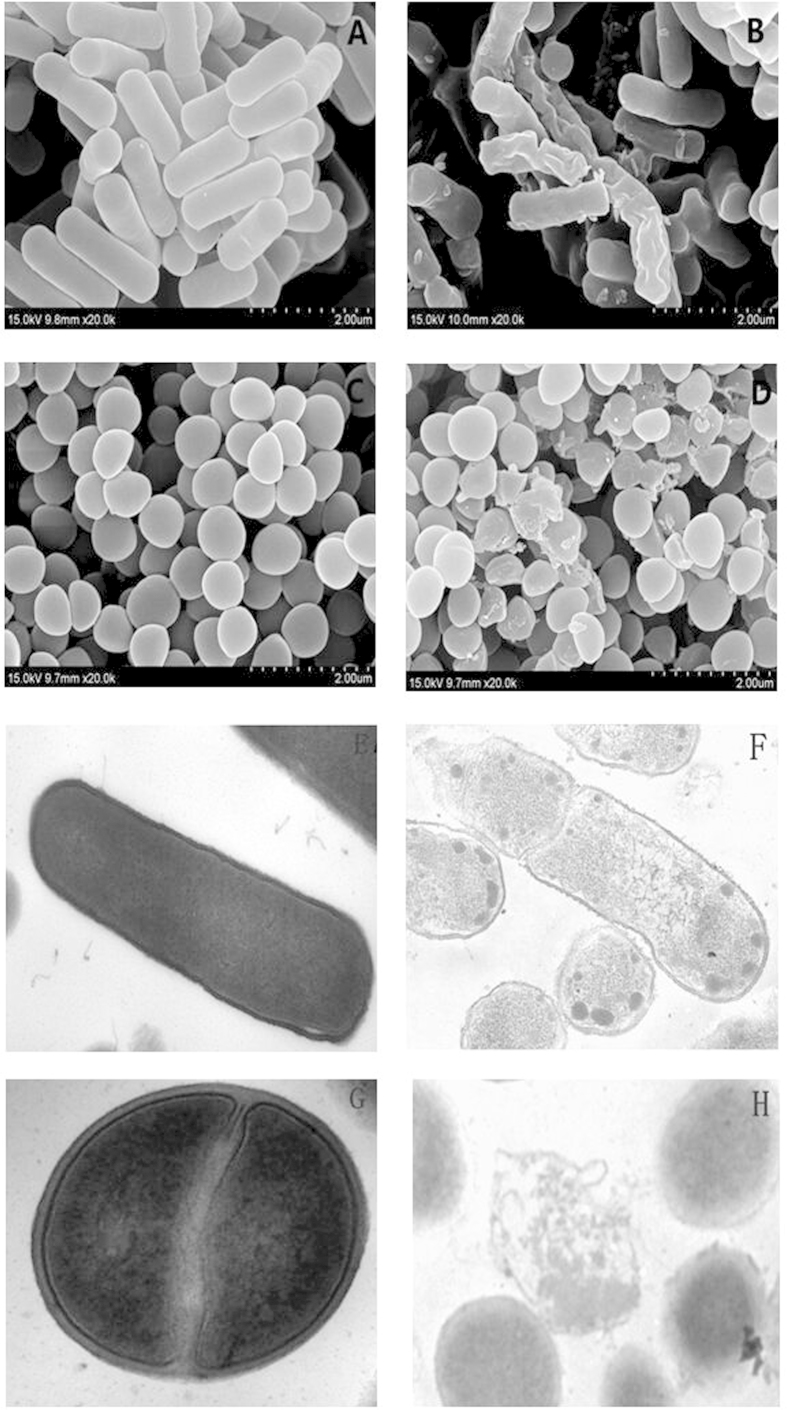
Scanning and transmission electron micrographs of *E. coli* 25922 and *S. aureus* 29213 treated with the peptide dCATH. SEM micrographs of *E. coli*: (**A**) Control, no peptide; (**B**) peptide-treated. SEM micrographs of *S. aureus*: (**C**) Control, no peptide; (**D**) peptide-treated. TEM micrographs of *E. coli*: (**E**) Control, no peptide; (**F**) dCATH-treated; TEM micrographs of *S*. *aureus*: (**G**) Control, no peptide; (**H**) dCATH-treated. Bacteria in mid-logarithmic phase were treated with the peptide at 1× MIC for 1 h.

The effect of the peptide dCATH on the membrane integrity of *E. coli* and *S. aureus* was examined. In the control without peptide treatment, all cells displayed a plump shape and smooth surface (Fig. 7A,C). The membrane surface of peptide-treated *E. coli* cells became shrunken, and cell morphology was changed (Fig. 7B). The effect of the peptide on *S. aureus* is shown in Fig. 7D, and the cell surface became obviously roughened and disrupted when treated with the peptide.

### Transmission electron microscope (TEM)

The morphology and intracellular alteration of bacterial cells treated with dCATH at 1× MIC were also visualized using TEM. In the control without peptide treatment, *E. coli* and *S. aureus* cells showed a smooth surface and dense internal structure, and the cytoplasmic content was evenly distributed and filled the whole space encapsulated by the bacterial wall. However, after 60 min of treatment with dCATH, significant rupture of the *E. coli* cell membrane and the release of cellular contents were observed (Fig. 7F). Furthermore, as compared with the control, the peptide induced significant changes in membrane morphology and intracellular alteration in *S. aureus* cells (Fig. 7H). Treatment with dCATH caused the collapse of the plasma membrane and dispersion of intracellular contents. Besides acting on the cell membrane, the peptide dCATH also caused the cytoplasmic components of *E. coli* cells to be condensed (Fig. 7F), which may imply a different action mechanism from membrane destabilization.

## Discussion

With the increase in the incidence of antibiotic-resistant bacteria worldwide, the need to develop novel classes of antimicrobial compounds to fight infectious diseases also increases^18^. AMPs have attracted a great deal of attention as alternative antibiotic candidates because of their broad spectrum of activity against Gram-positive and Gram-negative bacteria, enveloped viruses including HIV, and fungi such as *Candida* and *Cryptococcus*^19^,^20^. The AMP family of cathelicidins has roles in wound repair, inhibition of tissue damage when combined with endotoxin, cell chemotaxis and angiogenesis promotion, among other important activities^21^.

The cathelicidin family was first recognized in 1993^7^ and consists of numerous members that are distributed among various species. Most of these congeners have been identified through molecular biological approaches based on the high conservation of their prosequence^9^,^10^,^11^,^12^,^13^,^14^. These effector molecules of innate immunity are peculiar in that a highly conserved proregion is associated with a highly variable and biologically active unit. In the present study, a novel cathelicidin was discovered from ducks using the RACE-PCR method. This PCR reaction, which used primers based on a conserved sequence in the cathelin domain, did not amplify other cathelicidin products, suggesting that only one cathelicidin is expressed in Shaoxing ducks. This identification of a single cathelicidin puts Shaoxing ducks in a class with other animals, such as humans, monkeys, dogs, rabbits, mice, rats and rainbow trout as “monocathelicidin species” that possess a only a single cathelicidin gene^6^,^22^.

As shown in Fig. 1, the dCATH cDNA encodes the conserved sequence of cathelicidin family, including the signal peptide and cathelin-like domain, which are common characteristics of animal cathelicidins, and a highly heterogeneous C-terminal antimicrobial domain. Additionally, dCATH contains four highly conserved cysteine residues.

It is known that proteolytic maturation is necessary for cathelicidin to manifest its antimicrobial activity^23^. This process is mediated by elastase in most of the cathelicidins, although exceptions exist; these exceptions are seen in human LL-37, which is cleaved by proteinase 3^24^, and rabbit p15s, which has no cleavage site for elastase so the unprocessed molecule contains all biological activity^25^,^26^. In the elastase-processed cathelicidins, the elastase-sensitive residue is typically a valine or an alanine or, in some cases, an isoleucine^27^. The processing enzyme responsible for cleavage remains unknown. However, it is generally believed that in fish, birds and mammals, the molecule is processed by elastase; valine is the most common elastase-sensitive residue^27^. Because there are two valines in the C-terminal region of the propeptide of dCATH, two potential mature peptides were synthesized chemically that could have been released in these two positions by elastase. The antibacterial activity of the peptide that was cleaved from valine117 (DSTMEPRQVKRFWQLVPLAIKIYRAWKRR) is very low (data not shown). Valine126 was confirmed to be the cleavage site of elastase, releasing the mature peptide composed of 20 amino acids (KRFWQLVPLAIKIYRAWKRR). When released to the environment by protease hydrolysis, mature peptides of the C-terminal end exert their antibacterial and immunomodulatory activities^6^.

From the homology contrast and phylogenetic tree analysis, we compared the precursor protein sequence of dCATH with other avian cathelicidins, and found very small differences between them, indicating that these proteins are closely related phylogenetically over the course of evolution. However, the mature peptide displays significant diversity, which may result from the different pathogenic microorganisms in different living environments. The signal peptide and cathelin-like domain of cathelicidins are extremely conserved across species, but the mature peptides at the C-terminal region are highly diversified even within a species.

In the natural AMPs, including cathelicidins, α-helical structure is the most universal^28^. In the current study, the secondary structure of the peptide dCATH in different solutions was analyzed using CD spectra. Structural changes in water (PBS) and membrane-like (SDS, TFE) environments are important for α-helical AMPs to interact with the bacterial cell membrane, including its antibacterial activity^29^. The CD spectra indicated that dCATH retained a random coil structure in sodium phosphate buffer, and changed to adopt typical α-helical structure in SDS and TFE, implying that dCATH possessed remarkable antimicrobial activity against a broad range of microbes, including Gram-positive bacteria and Gram-negative bacteria.

Hemolytic and cytotoxic activity of dCATH was evaluated by determining the damage and survival of normal cells upon treatment with the peptide. The cytotoxicity of dCATH was as active as fowlicidins^9^, but weaker than that of melittin at low concentrations. As shown in Fig. 5A, serum can reduce the cytotoxicity of the peptide. This is possibly attributed to the binding of serum albumin^30^. In addition, it is important that fetal bovine serum containing a large number of growth factors on the cell growth factor^31^.

One of the ways to kill bacteria by AMPs is via destroying the cell membrane^32^,^33^, and this also explains why AMPs do not promote drug resistance. In this study, membrane permeability was examined using microscopic techniques to further investigate the interaction between dCATH and the membranes. The outer membrane is an important part of the Gram-negative bacteria membrane and is mainly composed of negatively charged hydroxyl phospholipids^34^. NPN, a neutral hydrophobic fluorescent probe, is normally excluded by the outer membrane but exhibits increased fluorescence intensity when it partitions into the outer membrane. The experimental results showed that dCATH can destroy the cell membrane in a concentration-dependent manner (Fig. 5). The inner membrane permeability assay indicated that dCATH could permeabilize the cell inner membrane. The release of diSC_3_-5 caused by dCATH treatment demonstrated that the peptide caused the cell membrane to become polarized in a dose-dependent manner (Fig. 6). Inner membrane permeability and cytoplasmic membrane electrical potential experiments indicated that dCATH could cause an increase in inner membrane permeability, leading to a simultaneous dissipation in the electrical potential across the cytoplasmic membrane and cytoplasmic content leakage, thus leading to cell death.

The membrane permeability assays identified that the peptide is capable of destroying the bacterial cell membrane structure quickly, thus increasing its permeability. To further investigate the interaction between the peptide and membrane, SEM and TEM studies were performed. The results of SEM showed that dCATH caused membrane blebbing and withering due to leakage of fluid into the cytoplasm, suggesting that dCATH may destroy the cell membrane, causing membrane permeability change, and cytoplasm contents leakage. TEM studies also provided morphological evidence of the potent permeabilizing activity of the peptide. Taken together, these results indicate that the cationic peptide would selectively bind components of the cell membrane, and then change the membrane permeability of bacterial cells and destroying the membrane to kill bacteria.

However, the membrane mechanism of AMPs is not the only way to kill pathogenic microorganisms; some AMPs not only act on the cell membrane, but also enter the cells^33^. They can alter cytoplasmic membrane septum formation, inhibit cell-wall synthesis, inhibit nucleic-acid synthesis, inhibit protein synthesis or inhibit enzymatic activity^35^,^36^,^37^. In the cytoplasm of the Gram-negative bacteria *E. coli* 25922, the peptide dCATH originated production of high electronic dense granules (Fig. 7F). From the results obtained it can be postulated that dCATH would be able to interact with biological macromolecules and interfered with the normal metabolism of cells. But the detailed action mechanism of the peptide in *E. coli* cells needs to be further investigated.

In conclusion, we have identified a novel waterfowl cathelicidin peptide, dCATH, from ducks that offers new insights into the evolution of cathelicidins and the role of these host defense molecules in the innate defense system. Due to the secondary structure and expression patterns, dCATH belongs to the linear α-helical subgroup of cathelicidins found in humans, dogs, mice, and rabbits that possess a singe cathelicidin gene. dCATH has potent antibacterial activity against a broad spectrum bacteria while showing obvious hemolytic activity and cytotoxicity. The peptide dCATH exerts its antibacterial activity by destroying the cell membrane, thus resulting in cytosolic leakage and cell death.

## Materials and Methods

### RNA isolation, cDNA synthesis and RACE amplification

Total RNA was isolated from the marrow of a 2-week-old Shaoxing sheldrake (Shaoxing anatis) using RNAiso reagents (TaKaRa, Dalian, China). The experimental protocol was reviewed and approved by the ethics committee of the Hospital of Northeast Agricultural University. The method was carried out in accordance with the approved guidelines. RACE-PCR was employed to extend the cathelicidin cDNA gene sequence using a 3′-Full RACE Core Set (TaKaRa, Dalian, China) according to the manufacturer’s protocol. The first-strand cDNA was synthesized using PrimeScript Reverse Transcriptase by an oligonucleotide d(T) linked to an adapter sequence. Two upstream forward primers P1 (5′-AGGATGCTGAGCTGCTGGGT-3′) and P2 (5′-ATGCTGAGCTGCTGGGTGCT-3′) were designed based on the 5′-untranslated region and a highly conserved domain encoding the signal peptides of avian^9^,^12^,^13^, and at last were coupled with the downstream reverse primers (3′ RACE outer primer: 5′-TACCGTCGTTCCACTAGTGATTT-3′, 3′ RACE inner primer: 5′-CGCGGATCCTCCACTAGTGATTTCACTATAGG-3′). The nested PCR consisted of two runs using the same conditions: 94 °C for 5 min; 30 cycles of 94 °C for 30 s, 55 °C for 30 s and 72 °C for 60 s; followed by a final elongation step at 72 °C for 10 min. The PCR product was purified by gel electrophoresis and cloned into a pMD 18-T vector (TaKaRa, Dalian, China). cDNA sequences were analyzed using an ABI 3730 automated sequencer (Invitrogen) followed by a BLAST search (http://www.ncbi.nlm.nih.gov/).

### Phylogenetic tree construction

To investigate polypeptide similarity, we got some avian peptide sequences by NCBI BLAST search. Then the multi-sequence alignments were constructed using ClustalW (http://www.ebi.ac.uk/Tools/msa/clustalw2/). Based on the proregion and mature domain phylogenetic trees were constructed using the neighbor-joining method (Mega, version 4.0; www.megasoftware.net) by calculating the proportion of amino acid differences among all sequences.

### Peptide synthesis

The dCATH and melittin peptides used for bioactivity testing were synthesized using a GL Biochem peptide synthesizer (Shanghai Ltd., Shanghai, China). The purity was analyzed to be more than 95% by analytical reverse-phase high-performance liquid chromatography (RP-HPLC). Two peptides were dissolved in deionized water at a concentration of 2.56 mM and stored at −20 °C.

### Circular dichroism analysis

To investigate the secondary structure of the peptide dCATH in different environments, CD spectra of the peptide in different environments were obtained at 25 °C using a J-820 spectropolarimeter (Jasco, Tokyo, Japan). The peptide samples were resuspended in 10 mM sodium phosphate buffer solution (PBS), at either pH 7.4 (mimicking the aqueous environment), in 50% TFE (mimicking the hydrophobic environment of the microbial membrane) or at 30 mM SDS micellar concentration (mimicking a prokaryotic cell membrane with negative charge). The spectrum was obtained at a 10 nm/min scanning speed from 190 nm to 250 nm. The final concentration of the peptide was 150 mM. Three scans were collected, and an average was determined. The acquired CD signal spectra were then converted to mean residue ellipticity with the following equation:


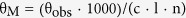


where θ_M_ is mean residue ellipticity (deg cm^2^ dmol^−1^), θ_obs_ is the observed ellipticity corrected for the buffer at a given wavelength (mdeg), c is the peptide concentration (mM), l is the path length (mm), and n is the number of amino acids.

### Antimicrobial assays

The minimal inhibitory concentrations (MICs) of the synthesized peptide were measured according to a modified version of the National Committee for Clinical Laboratory Standards broth microdilution method, as previously described^38^. Bacteria were incubated in Mueller Hinton (MH) broth to an exponential phase of growth and diluted with fresh MH broth to give a final concentration 1 × 10^5^ CFU/ml. Bacterial aliquots of 50 μl were distributed into a 96-well plate. Equal volumes (50 μl) of microorganism solution and 2-fold serially diluted concentrations of peptide in 0.01% (v/v) acetic acid and 0.2% (w/v) bovine serum albumin (BSA, Sigma) were added to each well of the sterile 96-well plate. After incubation for 24 h at 37 °C, MICs were determined as the lowest peptide concentration that resulted in no bacteria growth by optical density measurements (492 nm). The tests were performed in triplicate for each experiment.

### Hemolytic assay

The hemolytic activity of the peptide was measured as previously described^39^. Briefly, fresh anti-coagulated human blood was collected from a healthy donor (Harbin, China) in a polycarbonate tube containing heparin, The experimental protocol was reviewed and approved by the ethics committee of the Hospital of Northeast Agricultural University. (The method was carried out in accordance with the approved guidelines.) The blood washed twice with PBS, and then diluted to 1% in PBS with or without 10% FBS. Erythrocytes (90 μl) were assigned to a 96-well plate, followed by addition of 10 μl of serially diluted peptide with 0.01% acetic acid. The plate was centrifuged at 800 g for 10 min after incubation at 37 °C for 2 h. The supernatants were transferred to a new 96-well plate, and the absorbance was measured at 405 nm. Cells in PBS and 0.1% Triton X-100 were employed as negative and positive controls, respectively. Percent hemolysis was calculated as [(A_405 nm, peptide_–A_405 nm, PBS_)/(A_405 nm, 0.1%TritonX-100_–A_405 nm, PBS_)] × 100.

### Cytotoxicity assay

The colorimetric 3-[4, 5-dimethylthiozol-2-yl]-2, 5-diphenyltetrazolium bromide (MTT) dye reduction assay was used to determine the cytotoxicity of each peptide according to a previously described method^40^. Briefly, HaCat cells were diluted and placed into 96-well plates with a final concentration of 1.0 × 10^4^ cells/well in dulbecco’s modified eagle medium (DMEM). Peptides were added to the cell cultures to reach final concentrations of 1–128 μM. The cells were then incubated under a fully humidified atmosphere of 95% air and 5% CO_2_ at 37 °C for 18–24 h. After incubated with MTT (50 ml, 0.5 mg/ml) for 4 h at 37 °C, the cell cultures were centrifuged at 1, 000 g for 5 min, and the supernatants were discarded. Subsequently, 150 ml of dimethyl sulfoxide was added to dissolve the formazan crystals formed. Finally, the optical density was measured using a microplate reader (TECAN, Switzerland) at 492 nm.

### Outer membrane (OM) permeability assay

Outer membrane permeability activity was determined using the fluorescent dye NPN assay^41^. Briefly, *E. coli* UB1005 was grown to mid-log phase in MHB medium, washed three times with washing buffer (5 mM HEPES, 5 mM glucose, pH 7.4) and resuspended to 10^5^ CFU ml^−1^ in the same buffer. NPN was added to the bacteria at a final concentration of 10 mM, and the background fluorescence was recorded (excitation λ = 350 nm, emission λ = 420 nm). Fluorescence was determined by fluorescence spectrophotometer monitoring (TECAN, Austria). Peptide was added to a quartz cuvette to produce different final concentrations (0.5–8 mM). Fluorescence was recorded as a function of time until fluorescence no longer increased.

### Cytoplasmic membrane depolarization assay

The degree of change in cytoplasmic membrane potential was measured with the membrane potential-sensitive fluorescent dye diSC_3_-5 according to the previously described method^42^. *E. coli* UB1005 cells were grown to the mid-log phase at 37 °C, washed, and diluted to an OD_600_ of 0.05 in buffer (5 mM HEPES, 5 mM glucose, pH 7.4). The cell suspension was incubated with 0.4 mM diSC_3_-5 for 90 min so that most of the dye molecules gathered at the cytoplasmic membrane. The bacterial suspension was added to a 0.1 M final concentration of K^+^, and the cells were incubated at room temperature for 15–30 min. Membrane depolarization was measured after the addition of different concentrations of dCATH over a period of 600 s. Fluorescence was determined by fluorescence spectrophotometer monitoring (TECAN, Austria), with an excitation wavelength of 622 nm and an emission wavelength of 670 nm.

### SEM

As previously described^43^, *E. coli* ATCC 25922 and *S. aureus* ATCC 29213 cells were cultured to mid-log phase and harvested. Bacteria were washed twice with 10 mM PBS and resuspended to an OD_600_ of 0.2. The mixture of cells and the peptide (at a concentration of 1× MIC) was incubated at 37 °C for 60 min. The control was incubated without peptide. After being collected and washed with PBS three times, cells were fixed with 2.5% (w/v) glutaraldehyde at 4 °C overnight. The bacteria were washed twice with PBS to remove the fixed liquid and dehydrated through a graded ethanol series (50%, 70%, 90%, and 100%) for 15 min each. Upon dehydration, the dried bacterial cells were transferred to a 1:1 mixture of alcohol and tert-butanol for 30 min, followed by pure tert-butanol for 1 h. The samples were then dried using a critical point dryer. The dried bacterial specimens were coated and visualized under a field emission scanning electron microscope (HITACHI S-4800, Japan).

### TEM

Pre-treatment of bacterial samples was the same as for SEM, as described above. After pre-fixation with 2.5% glutaraldehyde, the bacterial pellets were washed twice with PBS and post-fixed with 2% osmium tetroxide for 80 min. Bacterial samples were washed twice with PBS, dehydrated for 8 min in each step of a graded ethanol series (50, 70, 90, and 100%), and incubated for 10 min each in 100% ethanol, a 1:1 mixture of 100% ethanol and acetone, and pure acetone. The samples were then immersed in 1:1 mixtures of pure acetone and epoxy resin for 30 min and in pure epoxy resin in a constant temperature incubator overnight. Finally, specimens were sectioned with an ultramicrotome, stained with uranyl acetate and lead citrate, and observed with a HITACHI H-7650 TEM.

### Statistical analysis

Data were analyzed by ANOVA using SPSS 16.0 software. Quantitative data are presented as the mean ± standard deviation of the mean. Differences were defined as significant at a P-value of less than 0.05.

## Additional Information

**How to cite this article**: Gao, W. *et al.* Identification of a novel cathelicidin antimicrobial peptide from ducks and determination of its functional activity and antibacterial mechanism. *Sci. Rep.* **5**, 17260; doi: 10.1038/srep17260 (2015).

## Figures and Tables

**Table 1:** MICs of the peptide dCATH against tested bacteria.

	MIC (μM)^a^	
Bacteria	dCATH	melittin
Gram-negative bacteria		
*Escherichia coli* ATCC25922	2	2
*Escherichia coli* UB1005	2	2
*Salmonella typhimurium* ATCC14028	4	2
*Salmonella pullorum* C79-13	8	4
Gram-positive bacteria		
*Staphylococcus aureus* ATCC29213	4	8
*Staphylococcus epidermidis* ATCC12228	4	0.5
*Enterococcus faecalis* ATCC29212	4	1
*Bacillus subtilis* CMCC63501	8	1
GM (μm)^b^	4	1.83

^a^Minimum inhibitory concentrations (MICs) were determined as the lowest concentration of the peptide that inhibited bacteria growth.

^b^The geometric mean (GM) of the MICs of the peptide against all bacterial strains was calculated.
